# Editorial: Hemispheric Asymmetries in the Auditory Domain

**DOI:** 10.3389/fnbeh.2022.892786

**Published:** 2022-04-06

**Authors:** Alfredo Brancucci, Nicole Angenstein

**Affiliations:** ^1^Dipartimento di Scienze Motorie, Umane e della Salute, Università di Roma “Foro Italico”, Rome, Italy; ^2^Combinatorial NeuroImaging Core Facility, Leibniz Institute for Neurobiology, Magdeburg, Germany

**Keywords:** hemispheric asymmetries, functional lateralization, auditory perception, functional connectivity, verbal cognition, musical cognition

The two cerebral hemispheres are specialized for different cognitive abilities and functional as well-structural asymmetries are present in many regions of the brain which control various types of functions, from sensory processing to motor activity (Brancucci and Tommasi, 2011; Corballis and Häberling, 2017; Westerhausen, 2019). The present issue focuses on *Hemispheric Asymmetries in the Auditory Domain*, a topic which has given rise to a huge amount of scientific work utilizing behavioral, neuropsychological, electrophysiological, and neuroimaging techniques (Bryden, 1963; Kimura, 1963; Brancucci et al., 2004, 2012; Prete et al., 2014, 2015, 2016, 2018a; D'Anselmo et al., 2016, 2018), possibly as auditory functions, as well as their underlying biological networks, are the most lateralized in the brain. The literature ranges from the study of the asymmetric processing during tasks on simple auditory stimuli (Brancucci and San Martini, 1999, 2003; Behne et al., 2005; Brancucci et al., 2005a; Brechmann and Scheich, 2005; Angenstein and Brechmann, 2013a,b; Brechmann and Angenstein, 2019), to the differential specialization of the hemispheres in the various aspects of language (Behne et al., 2006; Della Penna et al., 2007; Brancucci et al., 2008a; D'Anselmo et al., 2013; Wendt et al., 2021), to the latest evidence of asymmetries arising during social interactions (Brancucci et al., 2009). However, several points remain to be clarified, and there are still some unanswered questions, such as different plasticity processes in the two hemispheres especially with respect to clinical conditions, asymmetric functions of attention, and different asymmetric processes in language and music processing. The contributions to this Research Topic try to answer some of these questions, considering in particular aspects of auditory attention, plasticity processes in hearing impairment, the neural basis of language, and musical cognition.

Five papers focus on hemispheric asymmetries in the context of verbal processing (Kazimierczak et al.; Kljajevic; Moulin; Rødland et al.; Han et al.). In their fMRI study, Kazimierczak et al. looked for modulation of brain activity by top-down effects of forced-attention during a dichotic listening paradigm (Hugdahl, 2000). They observed the expected right-ear advantage. This behavioral effect was associated with a left-lateralized activity in speech perception areas of the temporal lobe. The directed forced attention to the left or right ear had the expected strong effects on the ear advantage. Unexpectedly, forced attention did not show a strong effect on brain activity neither with a traditional region analysis nor with a network analysis approach. They discuss their findings in relation to the classical theoretical models of dichotic listening and large scale cortical networks (Brancucci et al., 2005b, 2008b).

In an opinion paper, Kljajevic discusses different potential factors associated with sex differences in verbal learning (e.g., brain anatomy, sex hormones). The author emphasizes that sex differences in hemispheric asymmetry, namely planum temporale asymmetries, might be an important factor for the female advantage in verbal learning.

With a dichotic listening paradigm, Moulin showed that the word-level contextual influences during speech perception in hearing-impaired patients were stronger on the right than on the left ear. This asymmetry between the ears increased with increasing right-ear advantage and decreased with age, mainly due to an increasing contextual influence on the left ear. This age effect is consistent with reduced hemispheric asymmetry with age (see for example the HAROLD model, Cabeza, 2002).

Based on a visual language paradigm, Rødland et al. developed a short language paradigm for potential clinical application that can be used in the visual or auditory domain. Their fMRI-study showed that independent of the presented modality, the most central parts of the language system were activated by this paradigm mainly in the left hemisphere. Here, the connectivity patterns (revealed by dynamic causal modeling) were similar between presentation modalities whereas the right hemispheric involvement was different between modalities, e.g., more right frontal involvement during the visual than auditory condition.

In their paper on asymmetries in unilateral deafness, Han et al. start from the fact that unilateral deafness reduces the ability to localize binaural sounds and leads to a substantial change in auditory cortical processing. To unveil the underlying mechanisms, authors compared primary auditory cortical activity and hemispheric asymmetries of normal hearing, unilaterally deaf (right or left), and simulated hearing loss participants while listening to speech sounds delivered from different locations in the azimuth plane. The right ear hearing-loss group revealed slower reaction times for sound localization and prolonged neural latency compared to the left ear hearing-loss group. In addition, individuals of the right ear hearing-loss group with better sound localization showed increased responses in the hemisphere ipsilateral to the hearing ear. Conversely, the left ear hearing-loss group showed contralateral enhanced activity related to right ear stimulation. Thus, the authors demonstrated that right ear unilateral deafness can rely on better plastic reorganization compared to left ear deafness. This paper evidences the presence of potential plasticity targets, which should not be neglected in auditory rehabilitation.

Two other papers in this series look at hemispheric asymmetries in non-verbal processing (Kim et al.; Tanaka and Kirino). Tanaka and Kirino deal with a very interesting topic concerning musical imagery. They exploit the common condition of subjects who lie in an fMRI scanner and hear the typical machine noise using this condition as a baseline. During the musical imagery condition, in which musicians imagined to perform a music piece, they found enhanced right-lateralized connectivity of the auditory cortical areas with other brain regions devoted to cognitive, memory, and emotional processing. This outcome demonstrates that auditory cortex is involved with other areas in imagined music performance (Prete et al., 2018b).

Kim et al. investigate the intriguing issue of neural correlates of non-verbal syntax analyzing how syntactic irregularity and perceptual ambiguity on musical syntax are dissociated in terms of effective connectivity. Presenting three conditions in which a simple musical sequence ended with dominant- tonic, dominant-submediant or dominant-supertonic, they showed that left to right connectivity in bilateral inferior frontal gyrus was enhanced for the most irregular condition (dominant-supertonic) whereas connectivity from the right to the left superior temporal gyrus was enhanced for the most ambiguous condition (dominant-submediant). Thus, they showed that syntactic irregularity and perceptual ambiguity elicit dissociated processing between bilateral auditory areas.

The overall message that this Research Topic conveys is that *Hemispheric Asymmetries in the Auditory Domain* embrace competences that arise from various scientific fields whose interaction can bear fruit for cognitive neuroscience and for the other subject areas ranging around brain science. Exploiting the knowledge acquired on auditory hemispheric and functional asymmetries will generate further studies facing the question at different levels of analysis to disentangle the intriguing processes underlying such complex psychobiological functions.

## Author Contributions

All authors listed have made a substantial, direct, and intellectual contribution to the work and approved it for publication.

## Conflict of Interest

The authors declare that the research was conducted in the absence of any commercial or financial relationships that could be construed as a potential conflict of interest.

## Publisher's Note

All claims expressed in this article are solely those of the authors and do not necessarily represent those of their affiliated organizations, or those of the publisher, the editors and the reviewers. Any product that may be evaluated in this article, or claim that may be made by its manufacturer, is not guaranteed or endorsed by the publisher.
